# “Metabolic burden” explained: stress symptoms and its related responses induced by (over)expression of (heterologous) proteins in *Escherichia coli*

**DOI:** 10.1186/s12934-024-02370-9

**Published:** 2024-03-30

**Authors:** Sofie Snoeck, Chiara Guidi, Marjan De Mey

**Affiliations:** Department of Biotechnology, Centre for Synthetic Biology, Coupure Links 653, Gent, 9000 Belgium

**Keywords:** Stress response mechanisms, *Escherichia coli*, Protein expression, Metabolic engineering

## Abstract

**Background:**

Engineering bacterial strains to redirect the metabolism towards the production of a specific product has enabled the development of industrial biotechnology. However, rewiring the metabolism can have severe implications for a microorganism, rendering cells with stress symptoms such as a decreased growth rate, impaired protein synthesis, genetic instability and an aberrant cell size. On an industrial scale, this is reflected in processes that are not economically viable.

**Main text:**

In literature, most stress symptoms are attributed to “metabolic burden”, however the actual triggers and stress mechanisms involved are poorly understood. Therefore, in this literature review, we aimed to get a better insight in how metabolic engineering affects *Escherichia coli* and link the observed stress symptoms to its cause. Understanding the possible implications that chosen engineering strategies have, will help to guide the reader towards optimising the envisioned process more efficiently.

**Conclusion:**

This review addresses the gap in literature and discusses the triggers and effects of stress mechanisms that can be activated when (over)expressing (heterologous) proteins in *Escherichia coli*. It uncovers that the activation of the different stress mechanisms is complex and that many are interconnected. The reader is shown that care has to be taken when (over)expressing (heterologous) proteins as the cell’s metabolism is tightly regulated.

## Introduction

Global challenges such as climate change, maintaining biodiversity, depletion of fossil fuels and waste management have sparked social awareness of the importance of shifting towards a more bio-based economy [1, 2]. This shift includes unlocking the potential of renewable resources for the production of bio-based products and bioenergy [2]. These challenges have given industrial biotechnology a push, as it can provide a solution for many of these problems. Moreover, synthetic biology tools are increasingly emerging to facilitate metabolic engineering of strains and accelerate the development of industrial biotechnology processes [1, 2].

**Fig. 1:**
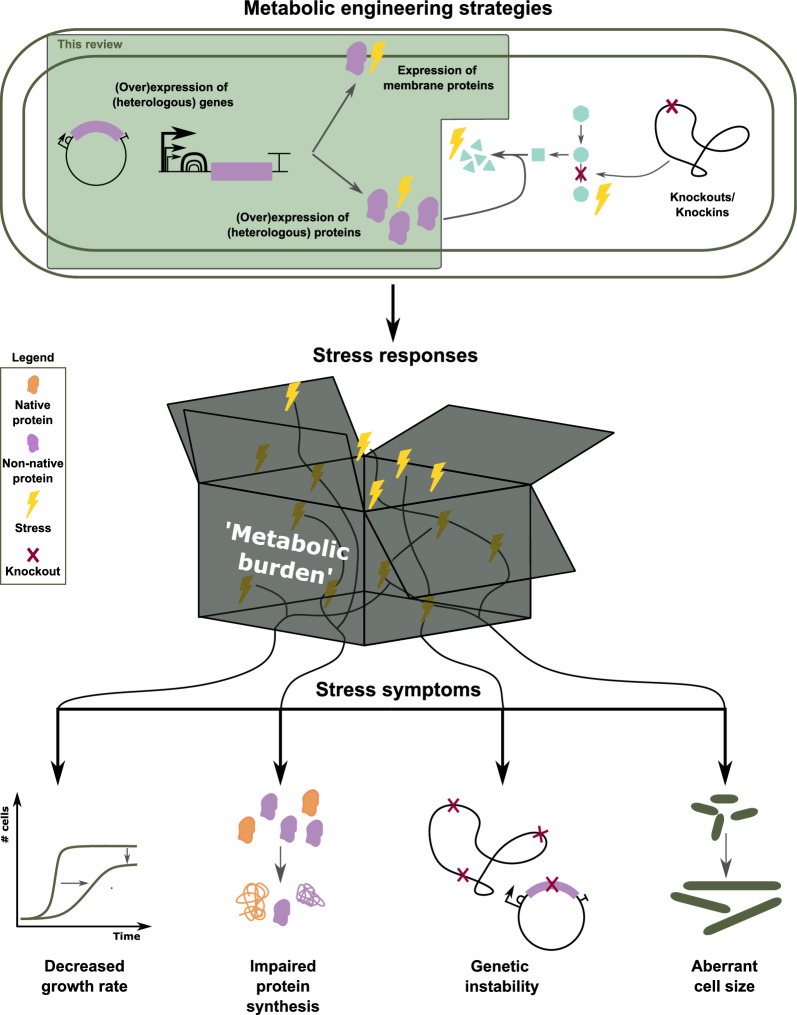
Schematic overview of the content of the review. Different metabolic engineering strategies often render stressed cells (top). This translates into different stress symptoms observed in the cells (bottom). However, the nature of the stress responses, their interconnections and translation in stress symptoms remains a black box (middle). This box is often summarised in the term “metabolic burden”, without specifying the associated responses. The focus of this review is to explore this black box, more specifically for the (over)expression of (heterologous) (membrane) proteins. Gene structures are displayed according to the SBOL guidelines [6]

Figure 1 (top) shows the most commonly used metabolic engineering strategies to redirect the metabolism towards the production of a specific product. (Over)expression of (heterologous) proteins increases catalysis of certain reactions or introduces non-native reactions to the host. Knockouts can remove side reactions that pull away precursors for the product of interest or the product of interest itself.

However, the host’s metabolism is complex as it has evolved towards a system that is highly regulated in such a way that cell growth and maintenance are benefitted [3]. It is therefore not straightforward to tweak the metabolism towards the (over)production of proteins or products, without disturbing the metabolic balance of the host organism. The proteins themselves can be stressful for the cell as well as the reactions they catalyse. Pathway engineering can lead to accumulation or depletion of (new) intermediates. This stress leads to diversification within the bacterial population [4] and will render cells with stress symptoms such as a decreased growth rate, impaired protein synthesis, genetic instability and an aberrant cell size (Fig. 1, bottom). On an industrial scale, these are reflected in low production titers and loss of newly acquired characteristics, especially in long fermentation runs [3, 5].

In literature these problems are being addressed extensively, but are always attributed to “metabolic burden”, which remains a black box (Fig. 1, middle). The actual connection between the cause of the stress and the stress symptom are rarely discussed. This review will uncover this black box of “metabolic burden”, firstly by giving an overview of how metabolically engineering cells can trigger different stress mechanisms and the intrinsic interconnectivity thereof. In a second part, the activated stress mechanisms will be linked to the most commonly observed stress symptoms. *Escherichia coli* is used as a model organism and the focus will be on stress caused by (over)expression of (heterologous) proteins and expression of membrane proteins, which trigger general responses. Moreover, stress mechanisms activated on the transcriptional level can be extended to the production of mRNA. When proteins are expressed from a plasmid, all stress mechanisms are relevant. Additional implications for the cells triggered by the plasmid itself are out of the scope of this review. Stress responses related to metabolites, including the accumulation or depletion of pathway intermediates as well as product toxicity will not be discussed in this review as these are very metabolite specific.

Even though this review focusses on *E. coli*, a lot of similar stress symptoms are seen for different host organisms, however, the exact regulation of stress mechanisms differs between organisms. This review can thus be used to get an understanding of the type of stress mechanisms that could be activated. The exact mechanisms activated in the specific host should then be further researched. The findings will be more applicable to closely related organisms, because of the more similar transcription/translation machinery, the structure of the cell wall etc. However, already having an idea of where to look for possible bottlenecks, will accelerate the development in other hosts as well.

These insights in “metabolic burden” will provide the reader with a more holistic view of metabolic engineering and its pitfalls. It will allow to identify the actual root of the problem and give the opportunity to prevent stress or relieve cells from their burden and render healthy cells with the desired characteristics. Understanding the connections between stress responses and their connection with stress symptoms will further accelerate industrial biotechnology applications. More specifically, it will give us insights on how to engineer the host’s metabolism without rupturing the cell viability. The specific solutions are out of the scope of this review, however, some examples will be given and the reader is referred to other literature for a more extensive discussion on this topic.

## (Over)expression of (heterologous) proteins

**Fig. 2:**
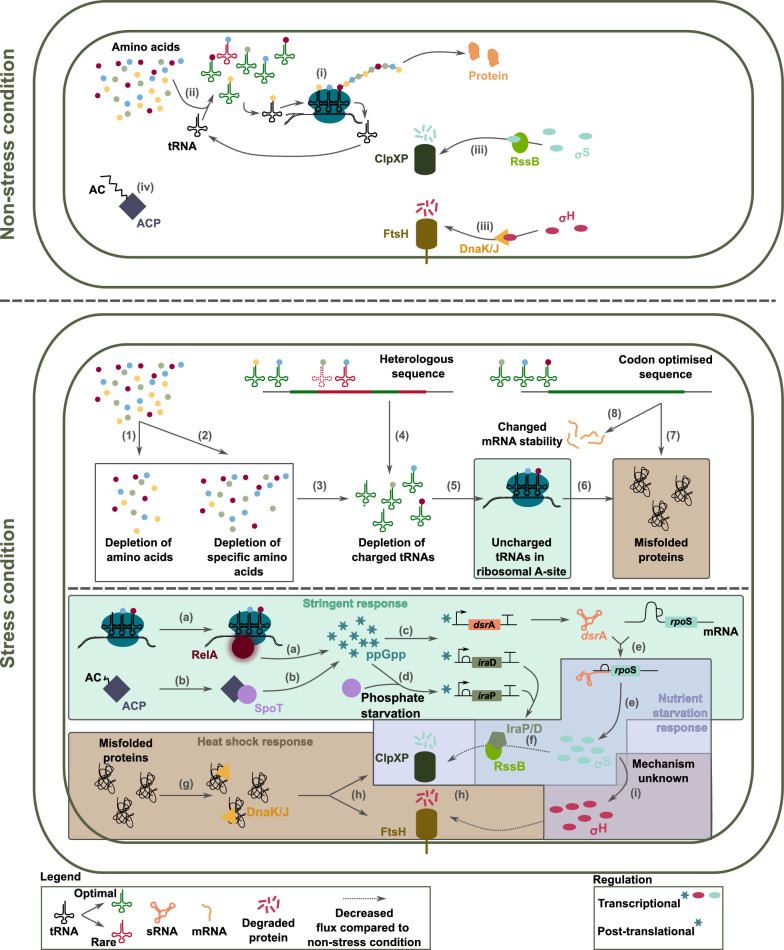
Overview of the stress responses induced by (over)expression of (heterologous) proteins. The top figure shows the situation in unstressed cells: (i) Protein synthesis; (ii) Charging of tRNA with their cognate amino acid; (iii) Degradation of $\sigma ^{\textrm{S}}$ and $\sigma ^{\textrm{H}}$ by ClpXP and FtsH respectively; (iv) AC bound to ACP. The bottom figure gives an overview of how (over)expression of (heterologous) proteins induces stress and how different stress mechanisms are connected. Effect on the cell: (1) Depletion of amino acids due to increased protein synthesis; (2) Depletion of specific amino acids because of a different amino acid composition; (3) Recharging of tRNAs compromised due to a lack of (specific) amino acids; (4) Depletion of tRNAs because of an over-use of rare codons; (5) Uncharged tRNAs present in the ribosomal A-site; (6) Translation errors occur due to long waiting times for the ribosome for the correct charged tRNA to arrive; (7) Codon optimised sequences result in misfolding of proteins; (8) Changed mRNA sequence influences its stability. Stress mechanisms: (a) RelA (stringent response) produces ppGpp in response to uncharged tRNAs in the ribosomal A-site; (b) SpoT (stringent response) produces ppGpp because of fatty acid starvation; (c) ppGpp transcriptionally activates several genes; (d) Transcription of *ira*P only happens under phosphate starvation in the presence of SpoT; (e) sRNA *dsr*A binds to *rpo*S, frees the RBS and allows translation; (f) IraP and IraD are anti-adaptor proteins, inhibiting the function of RssB to deliver $\sigma ^{\textrm{S}}$ to ClpXP for degradation; (g) DnaK/J are sequestered by misfolded proteins for refolding and degradation; (h) Proteases assist in degrading misfolded proteins, resulting in reduced degradation of $\sigma ^{\textrm{S}}$ and $\sigma ^{\textrm{H}}$; (i) $\sigma ^{\textrm{S}}$ involved in activating the heat shock response via an unknown mechanism. *ppGpp* guanosine tetra- and pentaphosphate, *AC* Acyl chain, *ACP* Acyl carrier protein, *RBS* ribosome binding site, *tRNA* transfer RNA, *mRNA* messenger RNA, *sRNA* small RNA. Gene structures are displayed according to the SBOL guidelines [6]

During protein synthesis, amino acids, the building blocks for proteins, are added to the growing amino acid chain by ribosomes via their cognate aminoacyl-tRNA [7, 8] (Fig. 2 (i)). Aminoacyl-tRNAs consist of anti-codons binding one or multiple codons and are charged with the corresponding amino acids (Fig. 2 (ii)). Levels of aminoacyl-tRNAs are in accordance with the codon usage of the organism [9, 10]. As aminoacyl-tRNAs for rare codons are less present, it will take longer for them to arrive to the ribosome and translation will slow down. The decoding time of rare codons can be up to three times higher, but is also dependent on the surrounding codons [11]. These regions are important for correct protein folding as it provides time for the proteins to fold [12–14].

In non-stress conditions, the chaperones DnaK and DnaJ bind free sigma factor H ($\sigma ^{\textrm{H}}$), inhibit its activity and make the sigma factor more accessible for degradation by FtsH [15]. Similarly, RssB binds sigma factor S ($\sigma ^{\textrm{S}}$) and delivers it to ClpXP for degradation [16, 17]. This ensures that levels of these alternative sigma factors are kept low (Fig. 2 (iii)). Acyl chains bind to the acyl carrier proteins (ACP) for among others fatty acid production (Fig. 2 (iv)).

### Depletion of amino acids and charged tRNA levels

Figure 2 (bottom) gives an overview of how the (over)expression of (heterologous) proteins (also depicted as non-native protein expression) can have an influence on amino acid and charged tRNA levels and induce different stress mechanisms. Firstly, (over)expressing a protein drains the pool of amino acids in the cell, also affecting native protein production [7, 8] (Fig. 2 (1)). Secondly, specific amino acids can deplete when the composition of the heterologous protein differs from the host’s innate proteins [18] (Fig. 2 (2)). Therefore, it is more difficult to find the desired amino acids to charge the cognate tRNAs, resulting in longer waiting times for the ribosomes or even uncharged tRNAs in the ribosomal A-site (Fig. 2 (3)). Thirdly, the codon usage is inherent to a microorganism, e.g., Leu is most commonly encoded by CUG in *E. coli*, whereas in *S. cerevisiae* UUG is preferred, and usage is correlated to cognate tRNA levels [9, 10]. Expressing a heterologous protein can thus lead to an over-use of rare codons, for which little cognate tRNAs are present (Fig. 2 (4)). This will have a similar effect as the depletion of amino acids, but here the lack of correct tRNAs and limited time to recharge tRNAs before the ribosome arrives at the next rare codon increases waiting times and uncharged tRNAs in the ribosomal A-site (Fig. 2 (5)) [12, 18–20]. If the waiting time for the cognate aminoacyl-tRNA to arrive at the ribosome is too long, translation errors, such as frameshifts, mutations or deletions of amino acids can occur leading to an increased amount of misfolded proteins (Fig. 2 (6)) [18, 19].

Codon optimisation, in which each codon of the original gene is replaced with the most abundant synonymous codon in the expression host, is often done to remove the discrepancy between the codon usage in the original host and the expression host. However, when sequences are codon optimised, rare codon regions originally present in the host disappear [12]. As mentioned above, rare codon regions can be important for correct protein folding by slowing down translation and providing time for the proteins to fold correctly [12–14]. Not taking these regions into account when transferring the desired gene to the host of interest can lead to misfolded proteins (Fig. 2 (7)) [11, 12]. Moreover, under amino acid starvation, tRNAs will be differentially charged to be able to maintain the expression of essential genes, which usually consist of optimal codons, creating direct competition between native and codon optimised genes [12, 21–23]. Furthermore, changing the nucleotide sequence of a gene also changes the mRNA sequence, which can severely impact mRNA secondary structure and thus influence stability and translation of the mRNA (Fig. 2 (8)) [12, 24]. The first stretch of the mRNA sequence has a major influence on translation initiation depending on how strongly the bases interact with each other [25].

### Stress responses associated with (over)expression of heterologous proteins

Expressing non-native genes can thus severely impact microbial cell factories, activating the stringent response due to amino acid/charged tRNA starvation [26, 27]. In addition, the translation errors increase the amount of misfolded proteins, which have reduced or no functionality. The pressure on chaperones and proteases in the cell rises, activating the heat shock and nutrient starvation response [18].

#### Stringent response

(Over)expressing (heterologous) proteins can severely impact amino acid and charged tRNA levels of the cells, which is a trigger for the activation of the stringent response. The alarmones guanosine tetra- and pentaphosphate (here collectively depicted as ppGpp) are the main actors in the stringent response [28]. ppGpp is synthesised by RelA in response to the presence of uncharged tRNAs in the ribosomal A-site (Fig. 2 (a)) [26, 27, 29]. A second enzyme SpoT is capable of both hydrolysing and synthesising ppGpp [30]. Its activity shifts towards synthesis under several nutrient limiting stresses. In case of fatty acid starvation, SpoT senses the fatty acid status of the cell through interaction with ACP, a cofactor involved in all processes of acyl chain synthesis [31, 32]. Indirectly, SpoT also senses carbon stress. Carbon starvation is accompanied by the depletion of acetyl coenzyme A (acetyl-CoA). Acetyl-CoA is the precursor for malonyl-CoA synthesis, which then interacts with ACP for further conversion into fatty acids [33]. Lower acetyl-CoA levels thus lead to lower malonyl-CoA levels, negatively impacting fatty acid synthesis and thus activating the synthesis activity of SpoT [31] (Fig. 2 (b)).

On a transcriptional level the stringent response regulates over 1000 genes either positively or negatively. ppGpp interacts with RNA polymerase (RNAP) and the transcription factor DksA for the regulation of the associated genes [34–36]. More in depth discussion of this regulation can be found in Gourse et al. [37] and Bange et al. [27]. The stringent response positively regulates amino acid biosynthesis, but has a negative effect on rRNA synthesis [27, 38]. Furthermore, genes related to nucleotide, RNA or protein (e.g., genes related to maturation, repair and proteolysis of proteins) metabolism and also DNA synthesis and translation are downregulated [27, 38, 39]. Additionally, ppGpp affects gene expression of genes involved in e.g., the plasma membrane and energy generation [38]. Many of these genes are involved in cell proliferation and growth and are mainly under control of $\sigma ^{70}$ [38, 40]. In general, there is a global reduction of transcription, which negatively influences supercoiling of the origin of replication, leading to a decrease in DNA replication [41]. Finally, ppGpp assists in DNA repair by enhancing RNAP pausing and backtracking [42, 43].

On a post-translational level, ppGpp binds many proteins involved in nucleotide metabolism (e.g., YgdH), ribosome biogenensis (e.g., initiation factor 2), maturation of dehydrogenases (e.g., HypB) and the metabolism of ppGpp (e.g., MutT) [44]. Due to its similarity to guanosine diphosphate (GDP) and guanosine triphosphate (GTP), ppGpp can occupy the binding site for these compounds in GTPases and block enzyme functionalities [27, 44]. In addition to blocking rRNA synthesis [27, 45], ppGpp interferes in every step of translation (initiation, elongation, termination and ribosome recycling), by blocking the actions of several factors involved in these processes [27]. Moreover, biogenesis and the maturation of the ribosomal subunits is reduced due to inhibition of the involved GTPases [27, 38, 44]. Every level of the translation process is thus negatively regulated by ppGpp.

Furthermore, the stringent response participates in the activation of the nutrient starvation response. The depletion of charged tRNA levels in the cells creates long waiting times for the ribosomes, increasing translation errors and thus misfolded proteins [18, 19], activating both the nutrient starvation and heat shock response [46, 47]. These connections are further elaborated on below.

#### Nutrient starvation response ($\sigma ^{\textrm{S}}$)

The nutrient starvation response, driven by the alternative sigma factor $\sigma ^{\textrm{S}}$ (encoded by *rpo*S), is activated in two ways when (over)expressing heterologous proteins. Firstly, ppGpp activates the transcription of *dsr*A [48, 49] and *ira*D [50] (Fig. 2 (c)). Under phosphate starvation and in the presence of SpoT, ppGpp also activates the transcription of *ira*P [51] (Fig. 2 (d)). *dsr*A, a sRNA, binds to the 5’ untranslated region (5’UTR) of *rpo*S, disrupts the internal loop and frees the ribosome binding site (RBS) for translation [49] (Fig. 2 (e)). *dsr*A contributes to the induction of *ira*D by translational repression of transcriptional regulator H-NS that negatively regulates those genes [52–54]. Both IraP and IraD are anti-adaptor proteins that inhibit the function of RssB by direct interaction, avoiding that RssB binds $\sigma ^{\textrm{S}}$ and delivers it to ClpXP for proteolysis [17, 50, 52] (Fig. 2 (f)). ppGpp thus regulates $\sigma ^{\textrm{S}}$ on a translational and post-translational level. Secondly, the depletion of both amino acid and charged tRNA levels and codon optimised sequences compromise correct folding of the proteins [13, 14]. As a consequence, there is an increase in misfolded proteins that have to be processed by proteases such as ClpXP (Fig. 2 (h)). ClpXP is thus pulled away towards the increased amounts of misfolded proteins in the cells and $\sigma ^{\textrm{S}}$ will be put in the waiting line. The cumulative effect of higher levels of ppGpp in the cells and queueing for proteases due to an increased production of misfolded proteins is responsible for the lack of degradation of $\sigma ^{\textrm{S}}$ and results in increased $\sigma ^{\textrm{S}}$ levels in the cell, activating the nutrient stress response [55].

$\sigma ^{\textrm{S}}$ activates the transcription of at least 500 genes [16, 56, 57]. Regulated genes span a wide range of functions connected to e.g., adaptation to stress, metabolism, transport and protein processing [58]. Moreover, $\sigma ^{\textrm{S}}$ has been shown to be involved in the switch between non-mutagenic and mutagenic double strand break repair [59–62].

#### Heat shock response ($\sigma ^{\textrm{H}}$)

The heat shock response is usually activated by elevated temperatures, but can also be triggered by misfolded proteins at low temperatures [46, 47]. $\sigma ^{\textrm{H}}$ is the sigma factor of the heat shock response and is encoded by *rpo*H. Regulation of the heat shock response happens on all levels (transcription, translation and post-translation). The response to the (over)expression of (heterologous) proteins and thus misfolded proteins mainly occurs on a post-translational level. In non-stress conditions, DnaK and DnaJ are the chaperones responsible for inhibiting $\sigma ^{\textrm{H}}$ activity and making the sigma factor more accessible to FtsH for degradation. Increased amounts of misfolded proteins sequester the chaperones for refolding and degradation, elevating levels of free $\sigma ^{\textrm{H}}$ (Fig. 2 (g)-(h)). The abundance of $\sigma ^{\textrm{H}}$ is thus regulated in a similar way as $\sigma ^{\textrm{S}}$, as their respective protease is overloaded in stressful conditions [55]. Recently, it has been shown that when mistranslation rates increase independent of a temperature upshift, the sequestration of proteases is not sufficient to fully activate the heat shock response [63]. Moreover, it was discovered that the activation is then dependent on $\sigma ^{\textrm{S}}$ (Fig. 2 (i)). The mechanism behind this is yet to be discovered [63].

The heat shock response increases the synthesis of heat shock proteins, which are mainly chaperones and proteases. These proteins help in the refolding or degradation of misfolded proteins to maintain protein homeostasis [64–66]. In addition, the $\sigma ^{\textrm{H}}$-regulated genes play a role in processes necessary for cell homeostasis under stressful conditions, including the homeostasis of complex proteins, preservation of DNA and membrane integrity [66–68]. Furthermore, $\sigma ^{\textrm{H}}$ regulates genes involved in modifying rRNA and tRNA, reducing DNA supercoiling, improving ribosome recycling and genes related to the central metabolism and transport. Finally, about a quarter of the genes regulated by $\sigma ^{\textrm{H}}$ are membrane proteins (MP) residing in the inner membrane of *E. coli*. This raises the possibility that the heat shock response also plays part in maintaining membrane homeostasis and clarifies why FtsH, its main degrader, is a membrane bound protein as well [66, 67].

## Expression of membrane proteins

**Fig. 3:**
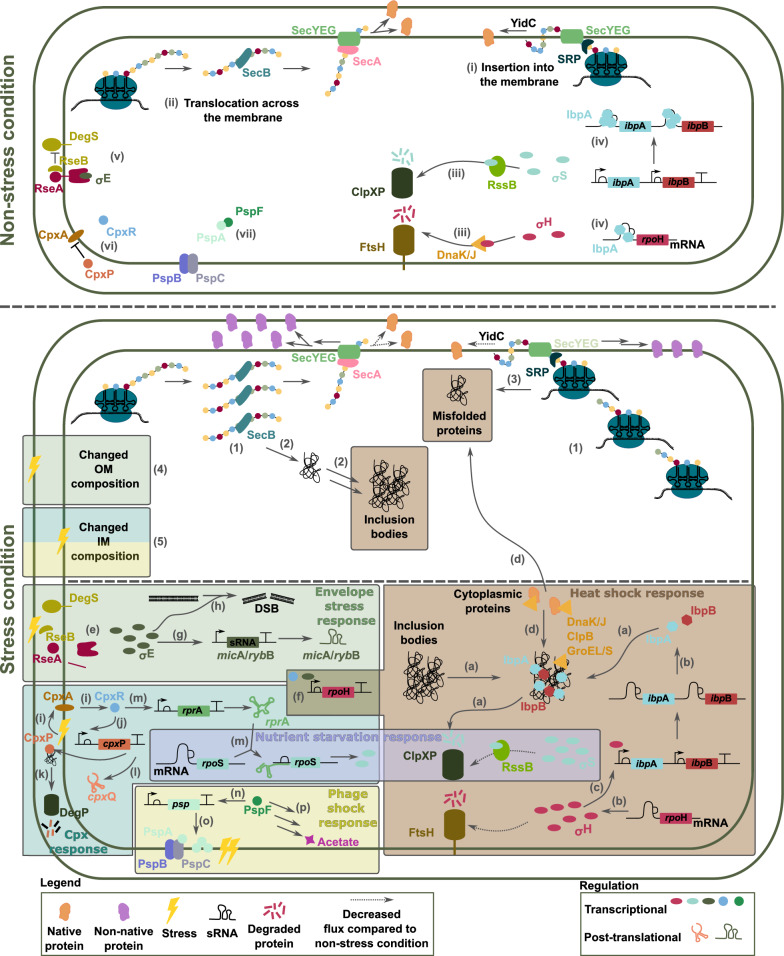
Overview of the stress responses induced by the expression of membrane proteins. The top figure shows the situation in unstressed cells: (i) Co-translational insertion of proteins into the membrane; (ii) Post-translational translocation of proteins across the IM; (iii) Degradation of $\sigma ^{\textrm{S}}$ and $\sigma ^{\textrm{H}}$ by ClpXP and FtsH respectively; (iv) Translational inhibition of *ibp*AB and *rpo*H by IbpA; (v) $\sigma ^{\textrm{E}}$ is kept inactive by RseA; (vi) CpxP prevents the activity of CpxA; (vii) PspA binds PspF to inhibit transcriptional activation. The bottom figure gives an overview of how expression of MP induces stress and how different stress mechanisms are connected. Effect on the cell: (1) Stalling of proteins that need to be inserted in or translocated across the IM; (2) Aggregation of secretory proteins and formation of IB; (3) Misfolding of IM proteins; (4) Stress at the OM due to changed composition; (5) Stress at the IM due to changed composition. Stress mechanisms: (a) Chaperones and sHSP co-aggregate with the IB for refolding and degradation; (b) Translational inhibition of *ibp*AB and *rpo*H by IbpA is lifted; (c) Transcriptional acitivation of *ibp*AB by $\sigma ^{\textrm{H}}$; (d) Sequestration of chaperones by IB induces misfolding of cytoplasmic proteins; (e) Release of $\sigma ^{\textrm{E}}$ by proteolysis of RseA; (f) Transcriptional activation of *rpo*H by $\sigma ^{\textrm{E}}$ and CpxR; (g) Activation of sRNAs *mic*A and *ryb*B; (h) Induction of DSB by $\sigma ^{\textrm{E}}$; (i) Activation of sensor kinase CpxA, activating the transcriptional regulator CpxR; (j) Activation of transcription of *cpx*P; (k) Sequestration of CpxP by misfolded proteins and degradation by DegP; (l) Transcription of the sRNA *cpx*Q; (m) Translational activation of *rpo*S by the sRNA *rpr*A; (n) Release of PspF and transcriptional activation of *psp* operon; (o) Positioning of PspA at the membrane and interaction with PspB an PspC; (p) Increased production of acetate via activation of different pathways.* ppGpp* guanosine tetra- and pentaphosphate,* IM* inner membrane,* IB* Inclusion bodies,* OM* outer membrane,* DSB* double stranded DNA break,* sRNA* small RNA,* sHSP* small heat shock protein. Gene structures are displayed according to the SBOL guidelines [6]

In the previous section the consequences of non-native protein expression in general were discussed. When the expressed proteins are located in the membrane, these responses are still activated, however, extra layers of complexity are introduced. Here, we will describe the stress responses related to MP (over)expression and elude how they are connected to or even aggravate the previously mentioned stresses. An overview of the stress responses that can be activated as a consequence of MP overexpression can be found in Fig. 3.

In *E. coli*, proteins that need to be inserted into or translocated across the inner membrane (IM) have an N-terminal signal sequence. If the signal sequences are hydrophobic they are usually recognised by a signal recognition particle (SRP) for co-translational insertion into the membrane [69, 70]. Ffh, the GTPase of SRP will form a complex with its membrane-associated receptor FtsY (also a GTPase) in a GTP dependent manner. GTP hydrolysis ensures stable binding to SecYEG (Sec translocon) and dissociation of SRP [71]. Finally, the Sec translocon together with YidC takes over the insertion into the IM (Fig. 3 (i)) [70–76]. Secretory proteins crossing the IM are guided to the Sec translocon by the ATPase SecA, sometimes with the additional help of the chaperone SecB [69–72, 74]. As SecA mediated translocation occurs post-translationally, SecB assists in keeping the amino acid chains unfolded as the SecYEG channel can only take unfolded proteins (Fig. 3 (ii)) [69–71]. Both the proteins heading for the IM, the periplasm or the outer membrane (OM) are thus in need of the Sec translocon. Next to the Sec translocon, the Tat pathway exports folded proteins across the IM [77]. However, the capacity of the Tat pathway is limited and only used for specific cases in metabolic engineering [78]. Therefore, it will not be considered further in this review.

In non-stress conditions, proteases ClpXP and FtsH keep levels of sigma factors $\sigma ^{\textrm{S}}$ and $\sigma ^{\textrm{H}}$ low (see section (Over)expression of (heterologous) proteins) (Fig. 3 (iii)). The small heat shock protein (sHSP) IbpA represses its own, *ibp*B and *rpo*H translation [79, 80] (Fig. 3 (iv)). When IbpA is bound to the *ibp*AB mRNA, it is degraded with the help of polynucleotide phosphorylase (PNPases) [79].

Sigma factor E ($\sigma ^{\textrm{E}}$) is kept inactive by the transmembrane protein RseA. RseB binds to RseA to prevent prevent proteolysis of RseA by DegS (Fig. 3 (v)) [81, 82]. CpxA is the sensory kinase of the CpxAR two-component system. CpxP is a periplasmic adaptor protein for CpxA and inhibits CpxA activity in the absence of stress (Fig. 3 (vi)) [82, 83]. Similarly, PspA binds to PspF to disable transcriptional activation (Fig. 3 (vii)) [84, 85].

### Traffic at the membrane

When overexpressing MP, these will also have to be inserted into or translocated across the IM by the Sec translocon. SecM senses the shortage of Sec translocon and will upregulate the expression of SecA by disrupting the secondary structure containing the Shine-Dalgarno sequence that stalls translation elongation. However in case of overexpression [74, 86], this upregulation is usually not enough for the translocation of all queuing proteins. The Sec translocon will be overloaded and proteins, both native and non-native, will be stalled at the membrane (Fig. 3 (1)). Secretory proteins have an aggregation prone signal sequence and if the unfolded sequences are stalled too long in the cytoplasm, they will aggregate in the cytoplasm and form inclusion bodies (Fig. 3 (2)) [74, 87]. MP will also misfold in the cytoplasm, but these are more readily degraded (ssrA tagged mRNA) (Fig. 3 (3)). Moreover, the cell envelope composition will change, as the Sec translocon is occupied with heterologous proteins and cannot insert or translocate all necessary native proteins [74]. This compromises the integritry of the outer membrane (OM) and IM (Fig. 3 (4)-(5)).

### Stress mechanism associated with membrane protein expression

As was mentioned for the (over)expression of (heterologous) proteins, expression of MP can also activate the stringent response, which is also interwoven with the nutrient starvation response (cfr. section Stringent response). The accumulation of misfolded proteins and the formation of inclusion bodies trigger the heat shock response and also the nutrient starvation response (cfr. section Nutrient starvation response (σ^S^) and Heat shock response (σ^H^)). Some extra layers of regulation are added here for the heat shock response. The insertion in and translocation of extra MP, has implications for the membrane integrity, which activates different membrane associated stress mechanisms such as the envelope stress response ($\sigma ^{\textrm{E}}$), Cpx response and the phage shock response.

#### Heat shock response ($\sigma ^{\textrm{H}}$)

The effect of misfolded proteins was already discussed in section Stress responses associated with (over)expression of heterologous proteins and is displayed in Fig. 2. Additionally, expression of MP increases the formation of inclusion bodies. sHSPs IbpA and IbpB will co-aggregate with the denatured proteins and facilitate refolding together with DnaK/J and ClpB (Fig. 3 (a)). This alleviates the translational repression of *ibp*AB and *rpo*H by IbpA (Fig. 3 (g)) and also stops mRNA degradation by PNPases [79]. The increased translation and reduced degradation of $\sigma ^{\textrm{H}}$ further increases *ibp*AB transcription, as it is transcriptionally activated by $\sigma ^{\textrm{H}}$ [79] (Fig. 3 (c)).

The sequestration of chaperones by misfolded proteins and inclusion bodies, decreases their availability for native proteins that need chaperones for proper folding (Fig. 3 (d)). As a consequence, not only proteins heading for the membrane, but also cytoplasmic proteins will misfold and aggregate. Accumulated proteins include the cell division protein MinD and the elongation factor Tu [74].

#### Envelope stress response

The (over)expression of MP leads to an overloaded Sec translocon. Consequently, there is less insertion in and translocation across the IM of native proteins. Secretory proteins aggregate in the cytoplasm, affecting the integrity of the OM (Fig. 3 (4)). Damage to the OM or the accumulation of OM proteins activates DegS, the protease responsible for the degradation of the $\sigma ^{\textrm{E}}$ anti-sigma factor RseA (Fig. 3 (e)) [81, 82]. RseB displaces from RseA in the presence of periplasmic lipopolysaccharides (LPS).

For full release of $\sigma ^{\textrm{E}}$, the activity of the transmembrane protein RseP and cytoplasmic protease ClpXP is needed.

$\sigma ^{\textrm{E}}$ activates the transcription of over 100 genes, mainly involved in cell envelope biogenesis (maintenance of LPS and OM protein levels) [81, 82, 88]. One of the targets is *rpo*H, thus inducing the heat shock response (Fig. 3 (f)) [88]. Furthermore, two sRNAs, *mic*A an *ryb*B, are transcribed, that are responsible for negative regulation of several genes (e.g., major porins) under envelope stress (Fig. 3 (g)) [88]. Finally, $\sigma ^{\textrm{E}}$, triggers double stranded DNA breaks (DSB) (Fig. 3 (h)) [89]. Next to damage of the OM, the stringent response can also activate the envelope stress response, via ppGpp that affects the activity of $\sigma ^{\textrm{E}}$ [90, 91].

#### Cpx response

The compromised insertion of IM proteins and translocation across the membrane, affect the IM composition and integrity, which is the main trigger of the Cpx response (Fig. 3 (5)). The triggers range from e.g., presence of misfolded MP [92], compromised peptidoglycan integrity [93] to impaired trafficking of lipoproteins [83]. Additionally, proteins that aggregate in the cytoplasm can also activate the Cpx stress response [94]. Activation of the sensory kinase CpxA can be mediated by the OM lipoprotein NlpE (other triggers also possible) [82, 83] and activates the DNA-binding response regulator CpxR (Fig. 3 (i)). CpxR induces the transcription of the CpxA adaptor protein CpxP (Fig. 3 (j)). However, CpxP is titrated away from CpxA by binding to misfolded proteins and degradation of CpxP and its substrate by DegP (Fig. 3 (k)). Once the stress is resolved, CpxP will be freed and inhibit CpxA activity [95]. Moreover, the 3’UTR of the *cpx*P mRNA codes for a sRNA, *cpx*Q, which is released by RNase E cleavage [96] (Fig. 3 (l)). Regulation of the Cpx response thus happens on a transcriptional, translational and post-translational level.

Genes coding for proteins carrying out protein folding and proteolysis in the cell envelope and cell wall modification enzymes are upregulated [92, 97]. Other target of CpxR are *rpo*H (Fig. 3 (f)) [98] and the sRNA *rpr*A [99] which, similarly to *dsr*A, binds to the 5’UTR of *rpo*S and induces translation (Fig. 3 (m)) [52, 100, 101]. The Cpx response is thus also linked to the heat shock and nutrient starvation response. Negative regulation mainly targets proteins involved in electron transport, oxidative phosphorylation, the tricarboxylic acid (TCA) cycle, transporters and iron or metal binding proteins [92, 97]. The sRNA *cpx*Q is responsible for the repression of a number of IM proteins [96]. The response ensures that the cell wall is not further overloaded with proteins and secondly that misfolded proteins are refolded or degraded.

#### Phage shock response

As for the Cpx response, damage to the IM causes activation of the phage shock response, however, this response needs more severe damage for activation (Fig. 3 (5)). One possible trigger for the phage shock response is the dissipation of proton motive force [84, 102]. The insertion of (heterologous) MP instead of native MP [74] changes the membrane composition with a reduction of respiratory chain complexes [74]. This is possibly reinforced by the activation of the Cpx pathway that reduces expression of proteins associated with electron transfer [92, 97], thus reducing the proton motive force. In case of inducing conditions, PspA will release the transcriptional activator, PspF, which activates the transcription of the *psp* operon and *psp*G, increasing the concentration of PspA compared to other Psp proteins (Fig. 3 (n)). PspA will preferably interact with PspB and PspC. These two proteins form an integral membrane complex that is thought to act as sensors for proton motive force stress (Fig. 3 (o)) [84, 85]. Both PspA as well as PspB and PspC are hypothesised to have a physiological role in the dissipation of the stress as global gene expression is not affected. However, more research is still needed to elucidate the working mechanisms [84, 85, 102].

The phage shock response is involved in the transition from aerobic to anaerobic respiration and ensures a reduction of processes requiring high energy, by activating genes like *arc*AB. The Arc two-component pathway activates the Pta pathway, resulting in an increased acetate production (Fig. 3 (p)) [74, 103]. Iron uptake and motility is downregulated, whereas cation import is upregulated. These conditions seem to direct the cell towards maintaining or restoring its proton motive force [104, 105].

## Stress symptoms

**Fig. 4:**
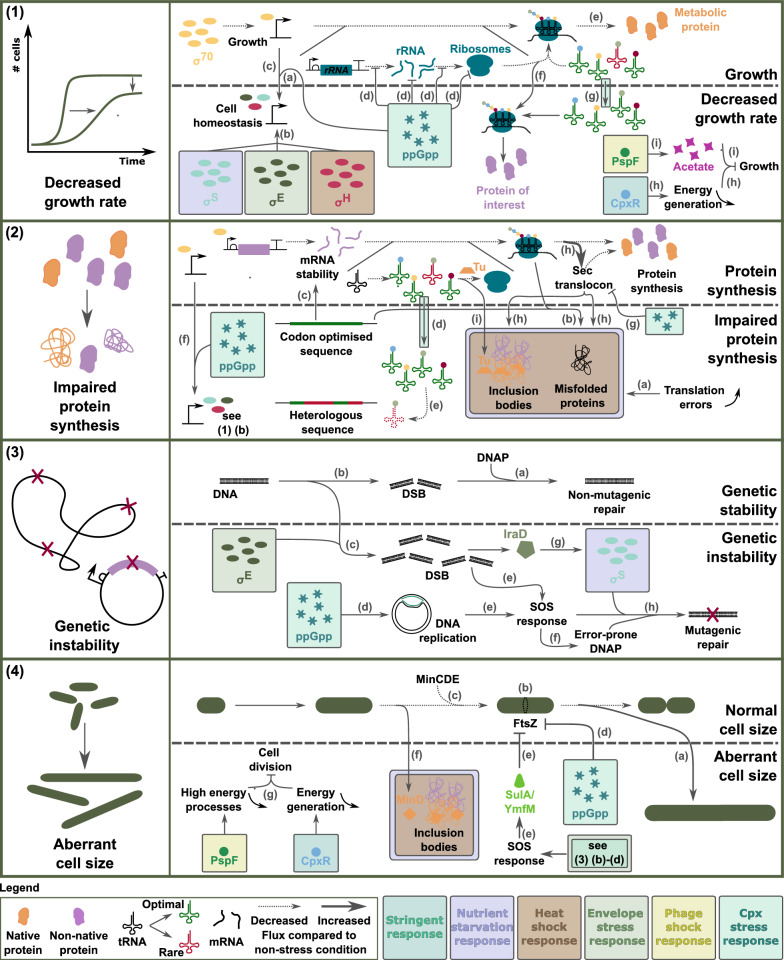
Stress mechanisms and cellular processes involved in the occurrence of stress symptoms when (over)expressing (heterologous) (membrane) proteins. **(1) Decreased growth rate**: (a) ppGpp destabilises $\sigma ^{70}$ promoter complexes and frees RNAP; (b) Increased amount of alternative sigma factors in the cell; (c) Increased transcription of genes dependent on alternative sigma factors; (d) ppGpp interferes in all processes of ribosome assembly and translation; (e) Less translation of metabolic proteins; (f) Translation of proteins of interest sequester part of the ribosomes; (g) Amino acid starvation induces differential charging of tRNAs, benefitting optimal codons; (h) Cpx response reduces energy generation which is negative for growth; (i) Phage shock response induces the production of acetate negatively impacting growth; **(2) Impaired protein synthesis**: (a) Increased translation errors lead to misfolded proteins; (b) Codon optimised sequences negatively impact folding of proteins and thus increase misfolded proteins; (c) Changing the mRNA sequence of genes changes their stability, which impacts translation; (d) Amino acid starvation induces differential charging of tRNAs, benefitting optimal codons; (e) The lack of rare codons negatively impacts expression of heterologous genes; (f) ppGpp together with increased amounts of alternative sigma factors decreases transcription from $\sigma ^{70}$ promoters and away from the gene of interest; (g) ppGpp inhibits GTPase activity, having a negative influence on insertion of proteins into the membrane; (h) Overloading of the Sec translocon has increased amounts of IB and misfolded proteins as a consequence; (i) Aggregation of elongation factor Tu, reduces the delivery of tRNAs to the ribosome. **(3) Genetic instability**: (a) Non-mutagenic DSB repair in unstressed cells; (b) Spontaneous DSB in growing *E. coli*; (c) $\sigma ^{\textrm{E}}$ increases the occurrence of DSB; (d) ppGpp inhibits DNA replication; (e) DSB and stalled DNA replication induce the SOS response; (f) Error-prone DNAPs are synthesised; (g) DSB induce *ira*D transcription and IraD stabilises $\sigma ^{\textrm{S}}$; (h) $\sigma ^{\textrm{S}}$ promotes the use of error-prone DNAP in DSB repair, increasing the mutation rate. **(4) Aberrant cell size**: (a) Filamentation of cells; (b) FtsZ locates mid-cell; (c) MinCDE assists FtsZ in localising mid-cell; (d) ppGpp inhibits GTPase activity of FtsZ; (e) SOS response activates cell division inhibitors SulA and YmfM; (f) MinD aggregates in IB, impacting cell division; (g) The Cpx response and the phage shock response negatively influence energy generation and high energy processes respectively, reducing cell division processes. $\sigma$ sigma factor,* ppGpp* guanosine tetra- and pentaphosphate,* RNAP* RNA polymerase,* IB *inclusion bodies,* rRNA* ribosomal RNA,* mRNA* messenger RNA,* tRNA* transfer RNA,* DNAP* DNA polymerase,* DSB* double stranded DNA break. Gene structures are displayed according to the SBOL guidelines [6]

As discussed, stress responses and their interconnectivity are complex and here they will be linked to specific stress symptoms that are often seen in metabolic engineering, i.e., decrease in growth rate and overall cell fitness, impaired protein synthesis, genetic instability and an aberrant cell size. An overview is displayed in Fig. 4. Furthermore, Table 1 gives some examples of (heterologous) proteins that were expressed and that lead to stress in *E. coli*. Identified stress mechanisms and possible mechanisms involved are given and if applicable the solutions applied by the authors as well.

### Decreased growth rate

When engineering microorganisms, one of the most commonly reported stress symptoms is a decreased cell fitness or growth rate (Fig. 4 (1)). Different aspects can play a role in this.

Firstly, elevated levels of ppGpp in combination with increased amounts of sigma factors, alter gene expression, disfavouring genes encoding for proteins important for cell proliferation and growth [38]. There have been a few theories in literature how this shift in gene expression is regulated and there seem to be multiple possible mechanisms [106]. One that is often mentioned is based on the availability of free RNAP and sigma factor competition [40, 107, 108]. The presence of ppGpp destabilises the open complex of promoters for genes involved in growth and cell proliferation (usually $\sigma ^{70}$ promoters), freeing the associated RNAP and thus increasing the availability of free RNAP (Fig. 4 (1) (a)). In addition, the amount of alternative sigma factors ($\sigma ^{\textrm{H}}$, $\sigma ^{\textrm{S}}$, $\sigma ^{\textrm{E}}$) in the cell increases, due to the joint effect of protease availability and changes in transcription (cfr. *ira*D) (Fig. 4 (1) (b)). Both the increased availability of RNAP core enzyme and the increased amount of alternative sigma factors in the cell, create an increased competitiveness of alternative sigma factors over $\sigma ^{70}$. This phenomenon accounts for the increased transcription from genes dependent on alternative sigma factors [40, 48, 107], that usually benefit cell homeostasis rather than cell growth (Fig. 4 (1) (c)) [58, 64–68]. Furthermore, high levels of ppGpp block rRNA synthesis and the maturation of ribosomes [27], decreasing the amount of ribosomes in the cell. Moreover, ppGpp interferes in all levels of translation (Fig. 4 (1) (d)) [27, 45]. This limited amount of ribosomes combined with a decreased translation means that less metabolic proteins, important for cell proliferation and growth, are produced (Fig. 4 (1) (e)). In addition some of the ribosomes available are occupied with the translation of recombinant proteins, being even less favourable for growth (Fig. 4 (1) (f)) [27, 45]. Furthermore, the expression of additional proteins drains part of the amino acids usually used for the synthesis of native proteins. Moreover, under amino acid starvation, tRNAs will be differentially charged to be able to maintain the expression of essential genes (and thus cell survival), which usually consist of optimal codons [12, 21, 22]. Codon optimised genes directly compete for these tRNAs, negatively influencing the production of proteins essential for growth (= metabolic proteins).

Membrane related stress responses have a negative impact on energy generation. The Cpx response downregulates genes connected to electron transport, the TCA cycle and oxidative phosphorylation [92, 97], meaning that less energy is being generated, negatively impacting growth (Fig. 4 (1) (g)). The Cpx response is also responsible for the increased translation of *rpo*S via the sRNA *rpr*A (Fig. 3 (m)) [99]. As mentioned above this shifts gene expression away from cell growth and proliferation.

Lastly, the phage shock response activates *arc*AB which enables the transition from aerobic to anaerobic respiration. As a consequence, acetate is produced as a byproduct [104, 105]. This compound is toxic and affects cell viability and growth of *E. coli* (Fig. 4 (1) (h)) [109, 110].

### Impaired protein synthesis

Often when (over)expressing (heterologous) genes, the protein levels found in the cell are not as expected. Proteins are either only present in very low numbers in the cells or the proteins are translated, but non-functional (Fig. 4 (2)).

(Over)expression of (heterologous) proteins increases the demand for tRNAs and amino acids, resulting in a higher amount of translation errors, especially when the codon usage or amino acid composition of the expressed proteins differ from the host’s. These translation errors together with compromised folding (especially in codon optimised sequences) render misfolded and thus non-functional proteins (Fig. 4 (2) (a)-(b)). A reduced amount of proteins can firstly be a consequence of adjusting the coding sequence of the desired proteins (e.g., codon optimisation). In addition to affecting folding of the protein, it also affects the stability of the mRNA (Fig. 4 (2) (c)) [12, 24]. Especially the first part of the sequence can severely impact translation initiation and thus impact the amount of translated protein [25]. Furthermore, differential charging of tRNAs under amino acid starvation to maintain the expression of essential genes (and thus cell survival), prioritises optimal codons (Fig. 4 (2) (d)) [12, 21, 22]. If heterologous genes contain many rare codons in their sequence, the lack of cognate tRNAs will negatively influence their expression (Fig. 4 (2) (e)). Finally, the increased amount of alternative $\sigma$-factors, which can occur under different stress conditions, and the presence of ppGpp shifts gene expression away from $\sigma ^{70}$ driven promoters (Fig. 4 (2) (f)) [40, 107, 108]. Most (over)expressed (heterologous) promoters are under control of a $\sigma ^{70}$ dependent promoter for stable and high production. The shift in gene expression will however result in less expression of the desired protein.

It was shown that ppGpp can compete with GTP for binding to GTPase and as such inhibit their activity [27, 38, 44]. If the stringent response is activated when expressing membrane proteins, ppGpp could possibly bind to the GTPase domain of SRP (Ffh) and its receptor FtsY. This will prevent the GTPase activity and thus the delivery of not only the desired MP [111], but overall insertion in and translocation across the IM could be even more severally impacted (Fig. 4 (2) (g)). In addition, overloading of the Sec translocon leads to queuing of secretory, IM and the desired MP at the cell membrane. Many of the proteins in the waiting line either aggregate in the cytoplasm or misfold and are degraded, some of the desired MP will thus never reach the IM (Fig. 4 (2) (h)). Moreover, cytoplasmic proteins aggregate due to the limited amount of chaperones and proteases available. Translation elongation factor Tu was found to co-aggregate with the secretory proteins [74]. This protein is responsible for the delivery of aminoacyl-tRNAs to the ribosome, a reduced amount of the elongation factor thus negatively influences the delivery of tRNAs (Fig. 4 (2) (i)). A lack of tRNAs triggers more translation errors and induction of ppGpp production, both negatively influencing the synthesis of the desired protein.

### Genetic instability

Metabolic engineering enables us to tweak cells to our specific needs, however, engineered cells often loose their desired traits due to mutagenesis, a process indicated as stress-induced mutagenesis (SIM) (Fig. 4 (3)). This genetic instability arises when mutations are introduced during DSB repair [59–62, 112]. In unstressed cells, mutations rarely occur during this process (Fig. 4 (3) (a)) [113], however, it can switch to more error-prone repair [59–62, 112].

Multiple components are involved in SIM. Firstly, in growing *E. coli*, per generation 1% of the cells accumulates at least one DSB (Fig. 4 (3) (b)) [114]. However, the envelope stress response, $\sigma ^{\textrm{E}}$, promotes double stranded DNA breaks, increasing this percentage (Fig. 4 (3) (c)) [89]. Moreover, ppGpp negatively influences DNA replication (Fig. 4 (3) (d)) [41], which just as the occurrence of DSB activates the SOS response (Fig. 4 (3) (e)). As a consequence an increased amount of error-prone DNA polymerases (DNAP IV and V) are produced (Fig. 4 (3) (f)) [59, 62, 113]. Finally, starvation conditions or (over)expression of (heterologous) genes, activate $\sigma ^{\textrm{S}}$, a key player in the switch between non-mutagenic and mutagenic DSB repair [59–62]. DSB also activate *ira*D expression, stabilising $\sigma ^{\textrm{S}}$ (Fig. 4 (3) (g)) [115]. $\sigma ^{\textrm{S}}$ promotes the use of DNAP IV, II and V in the repair of DSB, which more easily introduce mutations during the repair process (Fig. 4 (3) (h)) [59, 62, 116]. Stressed cells thus accumulate mutations and those beneficial for survival and growth will have an advantage. Therefore, the desired trait, that often is a burden for the cell, can get lost.

### Aberrant cell size

Another frequently observed symptom of stressed cells is the formation of filamentous cells (Fig. 4 (4)). Filamentation occurs when growth is continued yet cell division is inhibited, which protects the transmission of damaged DNA to daughter cells (Fig. 4 (4) (a)) [117, 118]. Filamentous cells can grow to a length of 10–50 times longer than normal *E. coli* [119, 120]. Division sites are still formed after the length of one cell is added to the filament. Therefore, cells can restart division at these sites when the stressor is removed and convert back to normally sized cells [120]. Different hypotheses of how (over)expression of (heterologous) (membrane) proteins can induce filamentation are discussed below.

When cells are about to divide, the protein FtsZ localises at the future division site and polymerises into a ring around the circumference of the cell (Fig. 4 (4) (b)). FtsZ is a self-activating GTPase and binding of GTP is required for its polymerisation. In addition, ZipA assists in anchoring FtsZ to the membrane after which other components needed for cell division are recruited [121, 122]. The proteins of the Min system aid in the positioning of FtsZ mid-cell (Fig. 4 (4) (c)) [123]. ATP bound dimeric MinD will bind to the membrane and will be able to interact with MinE and MinC. MinE stimulates the ATPase activity of MinD, releasing MinD from the membrane, this dynamic system creates a MinD concentration profile along the cell, with a minimum mid-cell. MinC is an inhibitor of FtsZ assembly and is recruited to the membrane by ATP-bound MinD. MinC will thus inhibit FtsZ assembly along the cell and ensure Z-ring assembly only occurs mid-cell [120, 123].

So far no connection has been found between cell division inhibition and increased ppGpp levels. However, GTP binding is crucial for the polymerisation of the FtsZ filament and its hydrolysis is hypothesised to help with the force generation during septation [121]. ppGpp could be involved in the inhibition of cell division as it can occupy the binding positions of GTP or GDP and inhibit enzyme functionality, avoiding Z-ring assembly (Fig. 4 (4) (d)) [27]. ppGpp levels do determine the size added to cells before division. Higher ppGpp concentrations that occur in response to several stresses lead to smaller added size [124]. This is counter intuitive, but if cell division is inhibited and filamentation occurs, the added size does not influence the length that cells can reach, as they will just not divide. Further research is needed to support this hypothesis.

In response to DNA damage or under influence of ppGpp/$\sigma ^{\textrm{E}}$, the SOS response is activated (see ’Genetic instability’). The latter activates the cell division inhibitors SulA and YmfM [125, 126]. These proteins inhibit the assembly of Z-rings by binding FtsZ, leading to filamentation (Fig. 4 (4) (e)) [117, 122].

Similar to elongation factor Tu, MinD aggregates in the cytoplasm when overexpressing MP (Fig. 4 (4) (f)) [74]. Overexpression of MinC induces filamentation in *E. coli*, but only in the absence of MinD. Sequestration of MinD in aggregates and the lack thereof at the membrane could have a similar effect. Finally, expression of MP can hamper the respiratory chain and thus cause problems with the energy state of the cell via the Cpx and phage shock response [74, 92, 97]. Cell divison requires a lot of energy so this defect could reduce the available ATP/GTP for Z ring assembly and anchoring of MinD to the membrane, hampering cell division (Fig. 4 (4) (g)) [122, 123].

**Table 1:** Overview of examples of (heterologous) protein production which lead to one of the above described stress symptoms (decreased growth rate, impaired protein production, genetic instability or an aberrant cell size)

Stress symptom	Engineering strategy	Possible mechanisms involved	Solution provided	References
Decreased growth rate	Plasmid-based inducible expression:	- Heat shock response	Dynamic expression of a sgRNA for target sequence under control of *hpt*G1 promoter identified to be upregulated	[127]
	- Reporter	- **Stringent response**		
	- Large heterologous proteins			
	- Operon encoding a metabolic pathway			
Decreased growth rate	Production of membrane proteins (GarP and YidC)	- Cpx stress response	sRNA based genetic circuit:	[94]
Impaired protein synthesis		- **Stringent response**	- Improved growth& protein production	
Aberrant cell size		- **Aggregation of MinD**	- Filamentation persisted	
Decreased growth rate	Production of membrane proteins (YidC, YedZ, LepI)	- Overloaded Sec translocon		[74]
Impaired protein synthesis		- **Phage shock response**		
Aberrant cell size		- **Aggregation of MinD**		
Impaired protein synthesis	Production of periplasmic proteins (Single-chain antibody fragment BL1)	- Overloaded Sec translocon	Lowering expression levels resulted in a decreased cell size & decreased amount of aggregates$\backslash$inclusion bodies	[87, 103]
Genetic instability		- Heat shock response		
Aberrant cell size		- **Phage shock response**		
		- **Envelope**$\backslash$**General stress response**		
Decreased growth rate	Expression of plant proteins	- Compromised protein folding	- Determining the amount of rare codons to select most suitable host	[13]
Impaired protein synthesis		- **Stringent response**	- Adding tRNAs of rare codons was detrimental to growth and protein solubility	
			**(Depletion of charged tRNAs and translation speed)**	
Genetic instability	Two synthetic genetic circuits (Induced by either AHL or IPTG)	- Recombination of repeated sequences	- Lower expression level to reduce metabolic load & thus mutation rate	[128]
		- **General stress response**	- Avoid use of repeated sequences in genetic circuits	

Stress mechanisms identified by the author are given and in addition our own input of stress mechanisms that could also be involved are indicated in bold

## General conclusion and discussion

This review highlighted that the term “metabolic burden” includes many different stress mechanisms that are highly interwoven. Traditionally, proteins are often expressed at the highest level possible believing this would result in the highest possible yield. However, these high expression levels can have severe consequences for the cell and completely abolish protein synthesis or functionality of the desired enzymes. It was shown that (heterologous) protein expression can activate multiple stress responses and how these result in the most commonly seen stress symptoms. Some stresses activate similar mechanisms and have the same stress symptom as an outcome. The cause of the stress should thus be correctly determined to apply the correct engineering strategy. Furthermore, care should be taken when expressing proteins, taking the cell’s metabolism and its limitations into account. Understanding the implications that (heterologous) protein expression can have on the cell is needed to prevent stress or to relieve engineered cells from stress. After identification of the bottleneck, existing engineering solutions can be implemented.

Methods exist to quantify the effect of (heterologous) (over)expression on the host’s cell health. For example, Ceroni et al. [127] developed the capacity monitor, which reacts to general metabolic burden. Furthermore, several biosensors exist to be able to quantify induction of more specific stresses [129]. Next to that, inducing the expression of proteins at a certain time point e.g., with the use of photogenetics [130] gives a clear view of the impact on the cells. On the other hand, prediction tools help to estimate e.g., the genetic stability of DNA sequences [131] or to simulate the effect of design parameters on genetic circuits [132].

Dynamic systems provide a very promising solution. This is a genetic circuit, that senses stress (e.g., activated stress response, accumulation/depletion of toxic product/intermediate) and inhibits the synthesis of the stressful protein or metabolite until stress is reduced. Other systems are based on quorum sensing or entry into the stationary phase and will split the growth and production phase, so that there is less competition between resources for the metabolic proteins and the desired proteins [133]. The cells thus self-regulate protein synthesis and related intermediates/products depending on their stress levels. Some examples are given in Guidi et al. [94], Dahl et al. [134] and Ceroni et al. [127].

When expressing heterologous genes, the sequence can be very important both for amino acid and tRNA depletion and for mRNA stability (see section (Over)expression of (heterologous) proteins). In contrast to codon optimisation, in codon harmonisation the gene of interest is adjusted to the codon usage frequencies of the native host. This ensures that proteins are folded more correctly [135] and avoids the over-use of certain tRNAs. mRNA stability can still be compromised and the use of certain codons (especially those that are commonly represented in highly expressed native genes [23]) can still cause problems as well as a different amino acid composition of the protein compared to the host can lead to depletion [18]. Therefore expression levels of the protein of interest can be adjusted to ensure resources are not limiting [136]. To this end, promoter/RBS libraries can be constructed or inducible promoters can be used.

Stress induced by (new) intermediates/products and the accumulation or depletion thereof was not discussed. Every metabolite has to be assessed individually to determine the appropriate actions. It should be kept in mind, that the metabolism is a complex web of reactions. Therefore, it might not always be the new product directly that causes stress, but cofactors or other reactions that need similar precursors as the new product might deplete or the product is converted into a toxic compound. Similarly, knocking out reactions can lead to downstream depletions, but also to the accumulation of toxic intermediates. Moreover, the localisation in the cell is important, as some metabolites will insert into the cell membrane and can cause membrane associated stress responses (e.g., free fatty acids [137]) as discussed in section Expression of membrane proteins. To conclude, when expecting stress from metabolites, the entire metabolism should be considered.

There is already a lot of research out there that focuses on reducing metabolic burden. This review will guide researchers to the correct solution for their problems, reducing the time needed to optimise biotechnological processes and render more stress resistant and productive strains. Above, we already hinted at a few possible solutions for stress relieve, for a full review on engineering strategies, we refer to other papers e.g., Boo et al. [8], T. Jiang et al. [138] and Perrino et al. [139].

This review gave an overview of how metabolic engineering (with a focus on protein expression) activates different stress mechanisms and how these lead to the stress symptoms that are often observed. More research is needed to verify some of the hypotheses outlined here, but it already provides a platform to further investigate on. A better understanding of why the engineered strains show certain symptoms will help to adapt engineering strategies to work more in sync with the bacterial cells and as such achieve the desired outcomes.

## Data Availability

Not applicable.

## Abbreviations

ppGpp: Guanosine tetra- and pentaphosphate
RBS: Ribosome binding site
ACP: Acyl carrier protein
MP: Membrane protein
OM: Outer membrane
IM: Inner membrane
SRP: Signal recognition particle
PNPase: Polynucleotide phosphorylase
sHSP: Small heat shock protein
LPS: Lipopolysaccharides
DSB: Double stranded DNA break
RNAP: RNA polymerase
DNAP: DNA polymerase
TCA: Tricaboxylic acid
SIM: Stress induced mutagenesis
